# Honeycomb Enhances the Egg-Laying Capacity of Laying Hens by Modulating Ovarian Function and Yolk Precursor Synthesis

**DOI:** 10.3390/ani16132016

**Published:** 2026-07-01

**Authors:** Shiji Zhu, Dengxu Zhu, Yukang Wu, Yuhao Zhang, Huiyu Wang, Yan Jiang, Wenwen Zhang, Qiang Cai, Wenju Liu, Shujuan Wang

**Affiliations:** 1 College of Animal Science, Anhui Science and Technology University, Chuzhou 233100, China; zshiji18133458327@163.com (S.Z.); zdx0114@126.com (D.Z.);; 2 College of Biomedicine and Health, Anhui Science and Technology University, Chuzhou 233100, China; 3 Yingshang County Center for Animal Disease Prevention and Control, Fuyang 236200, China; 4 Anhui Provincial Key Laboratory of Animal Nutritional Regulation and Health, Fengyang 233100, China

**Keywords:** honeycomb, production performance, intestinal health, ovarian function, yolk precursor

## Abstract

This study assessed the effects of dietary honeycomb supplementation on laying performance, intestinal inflammation, ovarian function, and yolk precursor synthesis in laying hens. A total of 320 Dawu Golden Phoenix laying hens (288-d-old) were randomly assigned into four treatment groups with eight replicates of 10 hens each. Hens were provided diets containing 0, 0.5, 1.0, or 2.0 g/kg honeycomb for 30 d. Dietary honeycomb significantly increased average egg weight, average daily feed intake, and laying rate, while decreasing the feed conversion ratio (*p* < 0.05). It enhanced serum antioxidant capacity, as reflected by higher GSH, SOD, and CAT activity (*p* < 0.05). Meanwhile, honeycomb modulated the expression of Nrf2/Keap1 pathway-related genes, accompanied by increased expression of downstream antioxidant-related genes. Additionally, honeycomb downregulated intestinal inflammatory- and apoptosis-related factors (*p* < 0.05), which effectively alleviated intestinal inflammation responses. In the liver, honeycomb showed favorable histological changes, including fewer lipid droplets and less vacuolar degeneration. Furthermore, it decreased serum ALT, AST, and TG levels and promoted yolk precursor synthesis by upregulating genes associated with lipid transport and vitellogenesis (*p* < 0.05). In the ovary, honeycomb optimized ovarian morphology and follicle development; elevated serum FSH, E2, and MLT levels; and enhanced the expression of steroidogenesis-related genes (*p* < 0.05), thereby promoting steroid hormone synthesis and reducing follicular atresia. In conclusion, dietary honeycomb supplementation could maintain intestinal health, modulate hepatic and ovarian metabolism functions, promote yolk precursor synthesis, improve follicle development, and consequently, enhance the laying performance of hens.

## Simple Summary

This study investigated the effects of dietary honeycomb on laying performance in hens. The results showed that honeycomb supplementation improved intestinal health and antioxidant capacity, accompanied by modulation of Nrf2/Keap1 pathway-related gene expression, enhanced liver lipid metabolism and yolk precursor synthesis, and promoted ovarian follicle development and reproductive hormone secretion. Overall, honeycomb can effectively improve egg-laying rate, egg weight, and feed efficiency and can be used as a natural feed additive to enhance the production performance of laying hens.

## 1. Introduction

The extension of the egg-laying cycle and the increase in egg production are crucial factors for reducing costs and improving economic benefits in the breeding of commercial laying hens. As laying age increases, the reproductive system gradually declines, accompanied by a reduction in the number of high-quality follicles [1]. In addition, long-term consumption of high-energy and high-protein diets leads to lipid accumulation. This promotes the generation of reactive oxygen species (ROS), weakens the total antioxidant capacity (T-AOC) and decreases the activities of antioxidant enzymes such as superoxide dismutase (SOD) [2,3]. This imbalance triggers oxidative stress, causing oxidative damage to granulosa cells and oocytes within the follicles [3]. Moreover, the transcriptional levels of reproductive hormone receptors and related genes decline, reducing steroid hormone synthesis and further accelerating ovarian dysfunction and follicular atresia [4]. These are the important factors that affect the decline of egg production performance of laying hens.

Previous studies have demonstrated that the decrease in egg production is closely associated with intestinal mucosal damage, reduced sex hormone levels, and insufficient synthesis of liver yolk precursors [5,6]. When ROS production and clearance are imbalanced, oxidative stress occurs, leading to ovarian dysfunction, diminished reproductive hormone levels, and depletion of the primordial follicle reserve [7]. Concurrently, inadequate nutrient absorption and impaired hepatic lipid metabolism reduce yolk precursor synthesis and deposition in the oocytes. These changes ultimately lead to a decline in egg production and associated economic losses [8,9]. During yolk precursor formation, lipids absorbed from the intestine are transported to the liver via the bloodstream. This is the predominant source of yolk precursor synthesis [10]. The synthesized yolk precursors are transported to the ovary through the synergistic action of microsomal triglyceride transfer protein (*MTTP*) and apolipoprotein B (*APOB*) [11]. In the liver, estradiol (E2) promotes histological and functional changes through the estrogen receptor-α (*ER-α*) signaling pathway, thereby promoting yolk precursor synthesis [12]. In addition, follicle-stimulating hormone (FSH) indirectly regulates yolk deposition by stimulating follicular development and modulating E2 and P4 levels [13]. Collectively, the intestine, liver and ovary interact through coordinated metabolite transport, hormonal signaling and molecular regulation, thereby directly affecting the laying performance of hens.

Green production modes for laying hens have become mainstream under the goal of carbon peaking and carbon neutrality, and research on antibiotic-free feed has attracted extensive attention. In recent years, numerous studies have confirmed that natural substances, plant extracts, and food processing by-products can enhance production performance and reduce feed costs by improving intestinal, ovarian, and liver functions in laying hens [14]. Bee products, including propolis, bee pollen, and royal jelly, are widely used in humans and animals [15]. As a by-product of bee products, honeycomb gradually increases with the growing market demand for bee products. Honeycomb is a natural by-product of the apiculture industry generated during beekeeping and honey harvesting. It contains residual honey, beeswax, flavonoids, polyphenols, and polysaccharides. Many of these components exhibit antioxidant and immunomodulatory activities. Currently, honeycomb is widely applied in food, medicine, and cosmetic industries, while its utilization is relatively limited in the animal husbandry industry. A previous study reported that dietary supplementation with honeycomb extract improved serum antioxidant capacity and immune function, while enhancing egg nutritional and flavor quality in laying ducks [16].

This study investigated the effects of dietary honeycomb supplementation on laying hens, with comprehensive measurements covering laying performance, egg quality, intestinal health, ovarian function, and liver yolk precursor formation. Although honeycomb contains abundant bioactive constituents, it is far less widely utilized in animal production compared when with royal jelly and propolis. To fill this research gap, the present experiment aimed to elucidate whether honeycomb could improve egg laying performance by regulating ovarian function and yolk precursor synthesis. Collectively, these results establish a theoretical basis for the application and popularization of honeycomb as a novel feed additive in the poultry industry.

## 2. Materials and Methods

### 2.1. Preparation of Honeycomb Samples

Honeycomb was purchased from Yinsong Beekeeping Farm (Fengyang, Anhui Province, China). The honeycomb was cut into cubes with a length of about 2–3 cm, dried at 50 °C for 48 h, cooled to room temperature, and subsequently frozen at −80 °C for 12 h. Then, the samples were crushed using a low-temperature crusher and passed through a 40 mesh sieve. The powdered honeycomb was stored at −20 °C for later use. The moisture content, total flavonoids, total polyphenols, and crude polysaccharides of the honeycomb were analyzed according to the method described by Chen et al. [16]. The honeycomb contained 7.01% moisture, 6.75 mg/g total flavonoids, 19.3 mg/g total polyphenols, and 4.84 mg/g crude polysaccharides. These results were used as the basis for supplementing honeycomb in the feed of the laying hens.

### 2.2. Animal Feeding and Management

The feeding experiment was conducted at Dingyuan Kangyuan Agricultural and Animal Husbandry Technology Co., Ltd. (Chuzhou, Anhui Province, China). A total of 320 healthy 288-d-old Dawu Golden Phoenix laying hens with similar weight and productive performance were selected and randomly divided into four dietary treatment groups, with eight replicates per group and 10 hens per replicate. The hens were housed in a well-ventilated enclosed poultry house equipped with tiered cages. Each bird was housed individually in a cage. Environmental conditions were maintained at 18–24 °C, and relative humidity was approximately 55–60%. A 16 h daily photoperiod was provided through artificial lighting.

At the beginning of the experiment, all groups exhibited a comparable laying rate of approximately 90%, with no significant differences observed among treatments. the control group was fed a basal diet, while the experimental groups were supplemented with 0.5 g/kg (H1), 1.0 g/kg (H2), and 2.0 g/kg (H3) of honeycomb added to the basal diet, respectively. The basal diet was formulated according to the laying hen feeding standards (NY/T 33-2004), with its composition and nutritional levels shown in Table 1. A 7 d adaptation period was arranged, followed by a 30 d formal feeding trial.

### 2.3. Sample Collection and Processing

On day 30 of the experiment, thirty eggs were randomly collected from each group for egg quality assessment. Laying hens with similar weight and good health were randomly selected from each group. The blood samples were collected from the jugular vein using vacuum blood collection tubes, and the serum samples were separated by centrifugation at 3500 rpm for 10 min at 4 °C, then stored at −80 °C for biochemical, antioxidant, and hormone analysis. Subsequently, the hens were euthanized by manual cervical dislocation and exsanguinated for tissue sampling. Samples of the duodenum, liver, and ovary were collected; rapidly frozen in liquid nitrogen; and stored at 80 °C for future analysis. Moreover, the liver and ovary were fixed in 4% paraformaldehyde for paraffin embedding.

### 2.4. Laying Performance Analysis

To assess the performance of egg production, the feed intake, leftover feed, egg count, and egg weight were recorded daily. The average daily feed intake (ADFI), feed-to-egg ratio (FCR), average lay rate (ALR), and average egg weight (AEW) were calculated using the following formulas:

ADFI (g/day) = total weekly feed consumption(g)/number of hens/days;

FCR (g/g) = total feed intake(g)/total egg weight(g);

ALR (%) = total number of eggs/number of laying hens alive/total number of days in the period × 100;

AEW (g) = total eggs weight(g)/total number of eggs.

### 2.5. Egg Quality Analysis

Egg quality parameters, including egg weight, Haugh unit, yolk color and albumen height, were assessed using a multifunctional egg quality tester (EMT7300, Robotmation Co., Ltd., Tokyo, Japan). Eggshell color was determined using a Chroma Meter (CR-400, Konica Minolta Co., Ltd., Tokyo, Japan). Eggshell strength was measured using an eggshell strength tester (RH-DQ200, Runhu Instruments Co., Ltd., Guangzhou, China). The pH values of the albumen, yolk and whole egg were measured using a pH meter. The maximum longitudinal and transverse diameters of the egg were measured with a digital caliper (14-648-17, Thermo Fisher Scientific, Waltham, MA, USA). The egg shape index was calculated as the ratio of maximum longitudinal diameter to maximum transverse diameter. Eggshell thickness was determined at the blunt end, equatorial region, and sharp end using an eggshell thickness gauge (ETG-1061, Robotmation, Tokyo, Japan). In addition, eggshell weight, yolk weight, and albumen weight were weighed separately after separation.

### 2.6. Serum Biochemical Parameters Analysis

Commercial assay kits provided by Nanjing Jiancheng Bioengineering Institute (Nanjing, China) were utilized to evaluate serum antioxidant indices and biochemical parameters, with a particular focus on antioxidant capacity (T-AOC; A015-2-1), glutathione peroxidase (GSH; A006-2-1), malondialdehyde (MDA; A003-1), superoxide dismutase (SOD; A001-1), and catalase (CAT; A007-1-1). The corresponding reagents were also used for the determination of serum biochemical parameters, including alanine aminotransferase (ALT; C009-2-1), aspartate aminotransferase (AST; C010-2-1), total cholesterol (T-CHO; A111-1-1), triglycerides (TG; A110-1-11), lipopolysaccharide (LPS; A054-2-1), and low-density lipoprotein cholesterol (LDL-C; A113-1-1). For all assays, blank, calibration, and sample wells were prepared in 96-well micro-plates, according to the manufacturer’s instructions. Serum samples (2.5 μL) were incubated with the corresponding reagents at 37 °C for the specified reaction time. Absorbance was recorded at the designated wavelength using a microplate reader (Thermo Fisher Scientific, Waltham, MA, USA). Antioxidant indices were calculated using calibration-based conversion factors provided by the manufacturer, whereas biochemical parameters were quantified using a single-point calibration method. All samples were analyzed in duplicate to ensure measurement reliability, and samples exceeding the linear detection range were appropriately diluted and reanalyzed before final data calculation.

### 2.7. Serum Hormones Analysis

Chicken-specific ELISA kits from Shanghai Bogu Bioengineering Institute (Shanghai, China) were utilized to quantify serum hormone profiles, including those for follicle-stimulating hormone (FSH; CHE052), progesterone (PROG; CHE063), luteinizing hormone (LH; CHE058), estrogen (E2; CHE050), and melatonin (MLT; CHE060). The detection sensitivities for FSH, PROG, LH, E2, and MLT were 0.1 ng/mL, 0.1 ng/mL, 0.1 ng/mL, 1.0 pg/mL, and 1.0 pg/mL, respectively. The corresponding detection ranges were 100 ng/mL, 25 ng/mL, 100 ng/mL, 2500 pg/mL, and 500 pg/mL, respectively. The intra-assay coefficient of variation (CV) was 10%, and the inter-assay (CV) was 15%. All samples were analyzed in duplicate to ensure the reliability of the measurements. Prior to the assay, the kits were equilibrated to room temperature for 30 min, according to the manufacturer’s instructions. Absorbance values were measured at 450 nm using a microplate reader (Thermo Fisher Scientific, Waltham, MA, USA).

### 2.8. Histological Analysis

Liver and ovarian samples were fixed in paraformaldehyde for 7 d, followed by routine dehydration, paraffin embedding, and sectioning into 5 μm thick slices. The prepared sections were subsequently subjected to hematoxylin and eosin (H&E) staining, according to the protocol reported by Wei et al. [17]. Histological examination and image acquisition were conducted using an automated digital slide scanning microscope (BA600-4, MOTIC, Xiamen, China).

### 2.9. Gene Expression Analysis

Total RNA was isolated from duodenum, liver, and ovarian tissues using TRIzol reagent following the manufacturer’s protocol (Accurate Biotechnology Co., Ltd., Changsha, China). The concentration and purity of the extracted RNA were determined with a NanoDrop spectrophotometer (Thermo Fisher Scientific, Waltham, MA, USA). Reverse transcription was performed using the 5× EvoM-MLV RT Reaction Mix Ver. 2 kit (Accurate Biotechnology Co., Ltd., Changsha, China). Real-time quantitative PCR (qPCR) was conducted using cDNA as the template, with gene expression levels detected by the SYBR Green dye method. The total reaction volume was 10 μL, consisting of 5 μL of 2× FastStart Universal SYBR Green Master Mix (Accurate Biotechnology Co., Ltd., Changsha, China), 1 μL of cDNA template, 0.5 μL of forward and reverse primers (10 μmol/L), and 3.5 μL of ddH_2_O. Amplification was performed using an FQD-96A Real-Time PCR System (Hangzhou Bioer Technology Co., Ltd., Hangzhou, Zhejiang, China). The thermal cycling conditions were as follows: initial denaturation at 95 °C for 5 min, followed by 45 cycles of denaturation at 95 °C for 5 s, annealing for 30 s, and extension at 72 °C for 20 s. A melting curve analysis was conducted at 60 °C for 25 s. Fluorescence signals were recorded at the end of each extension step and during the melting curve stage. Relative quantification of gene expression was calculated by comparing the cycle threshold (Ct) values of target genes with those of the reference gene. Primers were designed using Primer 5.0 software (PREMIER Biosoft, Palo Alto, CA, USA). All primers were synthesized by Sangon Biotech Co., Ltd. (Shanghai, China), and GAPDH was used as the internal reference gene (Supplementary Table S1).

### 2.10. Statistical Analysis

Relative mRNA expression was quantified by the 2^−ΔΔCt^ method. Statistical analyses were conducted using SPSS 26.0 (IBM Corp., Armonk, NY, USA) and GraphPad Prism 8.0 (GraphPad Software, San Diego, CA, USA). Differences among groups were evaluated by one-way ANOVA. Data are expressed as mean ± SEM, and statistical significance was defined at *p* < 0.05.

## 3. Results

### 3.1. Effects of Honeycomb on Laying Performance in Hens

The influence of dietary honeycomb supplementation on laying performance in hens is shown in Table 2. Dietary honeycomb supplementation significantly increased AEW, ADFI, and ALR in laying hens (*p* < 0.05), whereas no significant differences in ADFI and ALR were detected among the three supplemental treatment groups (*p* > 0.05). In addition, FCR was significantly reduced in the H2 group (*p* < 0.05).

### 3.2. Effects of Honeycomb on Egg Quality in Hens

The results of the egg quality assessment of hens fed with different levels of honeycomb for 30 d are shown in Table 3. Compared to the results for the control group, dietary supplementation with 2.0 g/kg honeycomb (H3) significantly increased eggshell color (*p* < 0.05). Additionally, eggshell thickness in the H1 group and eggshell weight in the H2 and H3 groups were significantly higher (*p* < 0.05), while the albumen weight was significantly increased across all treatment groups (*p* < 0.05). Yolk color was significantly reduced in the H3 group (*p* < 0.05). No significant differences were detected in the pH of whole eggs among the treatment groups (*p* = 0.062). The pH of albumen was significantly higher in the H2 and H3 groups, and the pH of yolk was significantly lower in the H2 group (*p* < 0.05).

### 3.3. Effects of Honeycomb on Serum Antioxidant Capacity in Hens

As shown in Table 4, dietary honeycomb supplementation significantly increased serum GSH content in the H3 group compared to that in the control (*p* < 0.05), while SOD and CAT activities were significantly elevated in all treatment groups (*p* < 0.05). The H2 group showed the most pronounced effects, whereas no significant differences were observed in total antioxidant capacity (T-AOC) and malondialdehyde (MDA) among all groups (*p* > 0.05).

### 3.4. Effects of Honeycomb on Serum Biochemical Parameters in Hens

Table 5 summarizes the serum biochemical parameters of laying hens receiving honeycomb supplementation. Compared with the control group, honeycomb supplementation significantly reduced serum ALT activity in the H1, H2, and H3 groups (*p* < 0.05). No significant differences were observed among the honeycomb groups (*p* > 0.05). AST activity was significantly reduced in the H2 and H3 groups, and the H3 group exhibited the lowest AST activity, which was significantly lower than that of all other groups. Serum TG levels were also significantly decreased in the H2 and H3 groups. No significant differences were observed in serum LPS, T-CHO, and LDL-C concentrations among any of the groups.

### 3.5. Effects of Honeycomb on Serum Reproductive Hormones in Hens

**Figure 1:**
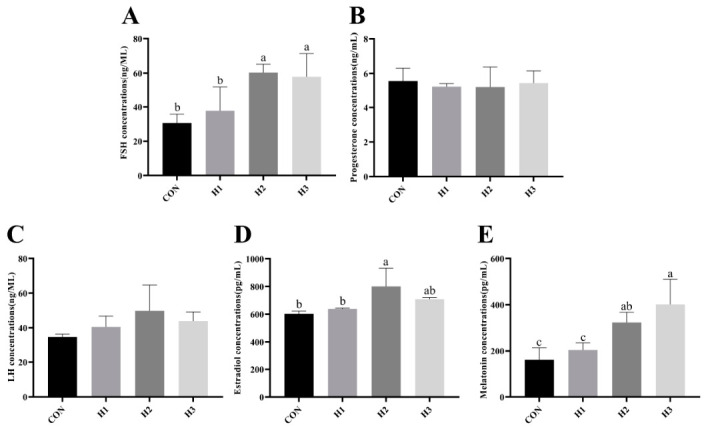
Effects of dietary honeycomb supplementation on serum steroid hormone levels in laying hens during the late peak production period. (**A**) Serum follicle-stimulating hormone (FSH) concentration; (**B**) Serum progesterone concentration; (**C**) Serum luteinizing hormone (LH) concentration; (**D**) Serum estradiol concentration; (**E**) Serum melatonin concentration. Values are presented as means ± SEM (*n* = 3). Values with different lowercase letters indicate significant differences (*p* < 0.05).

As shown in Figure 1, honeycomb supplementation significantly increased serum FSH concentration compared with the levels in the control group (*p* < 0.05). Similarly, serum melatonin concentrations were significantly increased in the H2 and H3 groups, with the highest concentration observed in the H3 group (*p* < 0.05). No significant differences were observed in serum P4 and LH concentration among all groups (*p* > 0.05). In addition, serum E2 concentrations were significantly increased in the H2 group compared with levels in the control and H1 groups (*p* < 0.05).

### 3.6. Effects of Honeycomb on the Expression of Genes Related to the Nrf2-Keap1 Signaling Pathway in the Intestine

**Figure 2:**
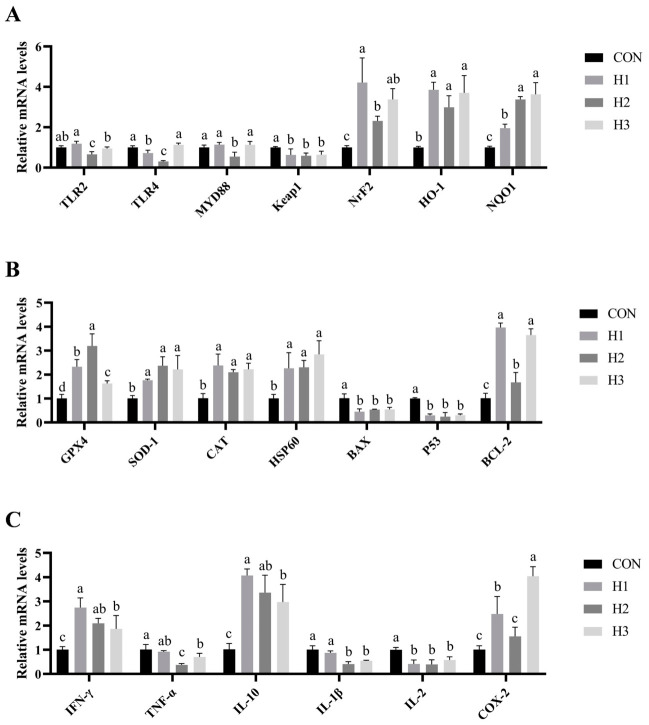
Effects of dietary honeycomb supplementation on intestinal function in laying hens during the late peak production period. *Nrf2-Keap1* signaling pathway-related gene expression (**A**), apoptosis and antioxidant-related gene expression in the intestine (**B**), and inflammation-related gene expression in the intestine (**C**). Values with different lowercase letters indicate significant differences (*p* < 0.05, *n* = 3).

As illustrated in Figure 2A, dietary honeycomb supplementation modulated the expression of Nrf2-Keap1 signaling pathway-related genes in the intestine of laying hens. Compared with the results for the control group, the expression of *Nrf2*, *HO-1*, and *NQO1* mRNA was significantly upregulated (*p* < 0.05), and *Keap1* mRNA was significantly downregulated in all treatment groups (*p* < 0.05). The expression of *TLR2* and *MYD88* mRNA in the H2 group and *TLR4* mRNA in the H1 and H2 groups was significantly reduced (*p* < 0.05).

### 3.7. Effects of Honeycomb on the Expression of Apoptosis- and Antioxidant-Related Genes in the Intestine of Hens

Figure 2B presents the effects of dietary honeycomb supplementation on the expression of apoptosis- and antioxidant-related genes in the hen intestine. The mRNA expression levels of antioxidant genes *GPX4*, *SOD-1*, and *CAT*, as well as the heat shock gene *HSP60*, were significantly upregulated (*p* < 0.05). In addition, compared with the control group, the expression levels of *BAX* and *P53* in all treatment groups were significantly decreased (*p* < 0.05), while the expression of *BCL-2* was significantly upregulated (*p* < 0.05).

### 3.8. Effects of Honeycomb on the Expression of Inflammation-Related Genes in the Intestine of Hens

The effects of honeycomb supplementation on the expression of intestinal inflammation-related genes in laying hens are shown in Figure 2C. Compared with the results for the control group, the expression levels of *IFN-γ* and *IL-10* were significantly upregulated in all treatment groups (*p* < 0.05); the expression of *COX-2* was significantly increased in the H1 and H3 groups (*p* < 0.05); the expression levels of *TNF-α* and *IL-1β* were significantly reduced in the H2 and H3 groups (*p* < 0.05); while the H1 group showed a nonsignificant decreasing trend (*p* > 0.05).

### 3.9. Effects of Honeycomb on the Expression of Genes Related to Yolk Precursor Synthesis in the Livers of Hens

**Figure 3:**
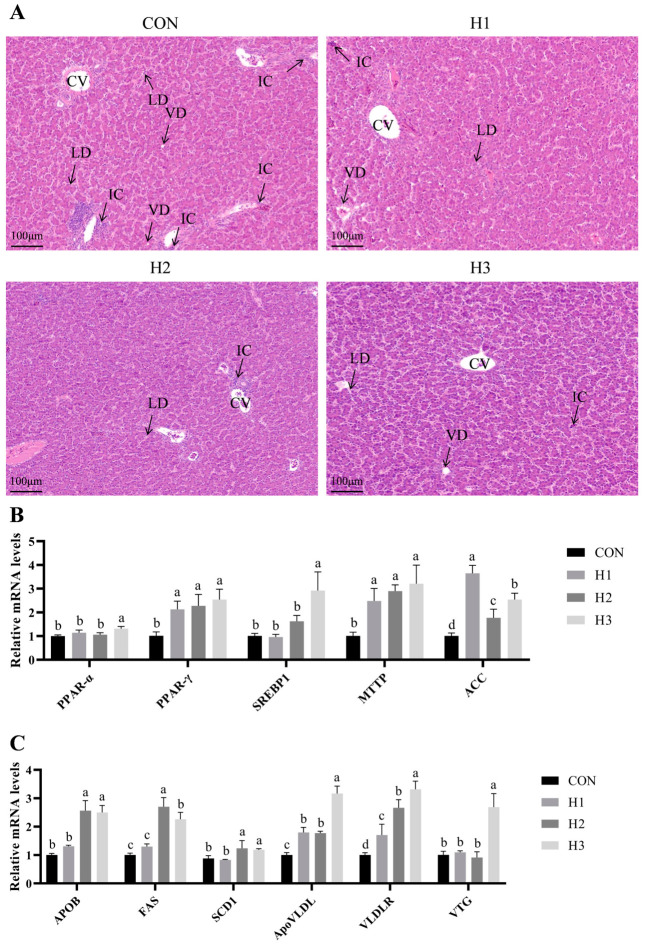
Effects of dietary honeycomb supplementation on hepatic function in laying hens during the late peak production period. Hematoxylin–eosin (H&E) staining of liver tissues (n = 4); scale bar = 100 μm (**A**). Expression of hepatic genes related to lipid metabolism and yolk precursor synthesis (*n* = 3) (**B**,**C**). Values with different lowercase letters indicate significant differences (*p* < 0.05). Abbreviations: CV, central vein; IC, inflammatory cell; LD, lipid droplet; VD, vacuolar degeneration.

Liver tissue morphology was observed by HE staining. The qualitative results showed that liver tissues in the control group exhibited evident lipid accumulation and inflammatory cell infiltration. There were fewer lipid droplets and less vacuolar degeneration in the livers of laying hens in the honeycomb-supplemented group. These histological qualitative observations indicate that honeycomb supplementation could alleviate hepatic lipid deposition and hepatic inflammatory responses (Figure 3A), indicating that honeycomb has a protective effect on liver tissue. In addition, the expression of genes related to the yolk precursor synthesis in the livers of laying hens was analyzed. The expression levels of *PPAR-γ*, *MTTP*, *ACC*, *ApoVLDL II*, and *VLDLR* in the honeycomb-supplemented groups were significantly upregulated (*p* < 0.05). *PPAR-α*, *SREBP1*, and *VTG II* levels in the H3 group were higher than those in the control group (*p* < 0.05). The H2 and H3 groups exhibited significantly increased expression levels of *APOB*, *FAS*, and *SCD* when compared to the levels in the control group (*p* < 0.05) (Figure 3B,C).

### 3.10. Effects of Honeycomb on the Expression of Genes Related to Follicular Development and Steroid Hormone Synthesis in Hens

**Figure 4:**
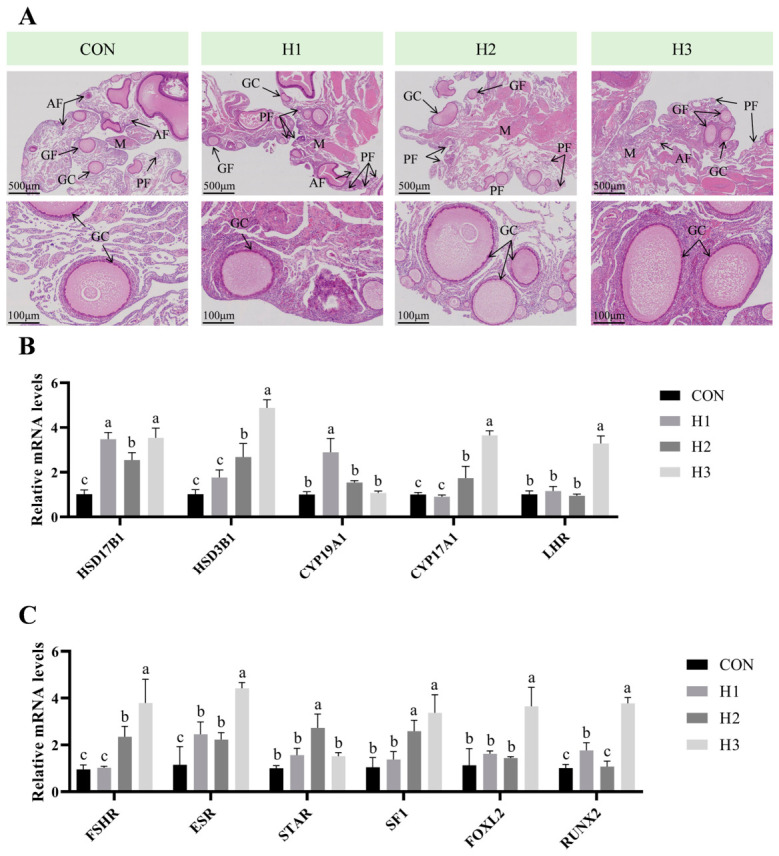
Effects of dietary honeycomb supplementation on ovarian function in laying hens during the late peak production period. Hematoxylin–eosin (H&E) staining of ovarian tissues (*n* = 4), with scale bars of 100 μm and 500 μm, respectively (**A**). Expression of ovarian hormone synthesis-related genes ((**B**,**C**), *n* = 3). Values with different lowercase letters indicate significant differences (*p* < 0.05). Abbreviations: AF, atretic follicle; GF, growing follicle; GC, granulosa cell; M, medulla; PF, primary follicle.

Ovary tissue morphology was observed by HE staining. The qualitative results showed that ovarian morphology and follicle count in the experimental group were superior to those in the control group, characterized by fewer atretic follicles, more growing and primary follicles, and a denser medullary structure. Based on the compactness of the thecal layer in mature follicles, it is speculated that honeycomb supplementation may promote granulosa cell development. These results indicate that honeycomb can improve ovarian tissue morphology and promote follicular development (Figure 4A). The expressions of steroidogenic genes in ovaries were analyzed. The expression levels of *HSD17B1* and *ESR1* in honeycomb-supplemented groups were significantly upregulated. The H2 and H3 groups showed significant upregulation of *HSD3B1*, *CYP17A1*, *FSHR* and *SF1*. The H1 group exhibited a significant increase in *CYP19A1* expression (*p* < 0.05), while *LHR* and *FOXL2* expression were significantly upregulated in the H3 group (*p* < 0.05). Additionally, *STAR* expression in the H2 group was significantly upregulated (*p* < 0.05), while *RUNX2* mRNA expression was markedly increased in the H1 and H3 groups (Figure 4B,C, *p* < 0.05).

## 4. Discussion

Maintenance of laying performance in hens depends on efficient intestinal nutrient absorption and the hepatic synthesis of yolk precursor substances [1,9]. These precursors play a key role in yolk deposition, follicular growth, and maturation [18]. Insufficient yolk precursor supply can impair follicular development and reduce egg production and egg quality [19]. Therefore, nutrition supporting intestinal, hepatic and reproductive functions are important for maintaining the productivity of laying hens. The present study explored the effects of honeycomb supplementation on laying performance, antioxidant status, intestinal health, hepatic metabolism and ovarian function in laying hens. These findings indicated that honeycomb supplementation exerted beneficial effects across multiple physiological systems rather than through a single mechanism. Improvements in antioxidant status and intestinal health may enhance nutrient utilization, whereas enhanced hepatic metabolism and ovarian function may promote yolk precursor synthesis and follicular development. Together, these coordinated responses may contribute to the improved laying performance observed in honeycomb-supplemented hens.

Maintaining high production performance is essential for maximizing economic benefits in the egg industry, with egg production rate and egg quality regarded as core evaluation indicators. In the present study, dietary supplementation with honeycomb increased average egg weight, feed intake, and egg production rate and significantly reduced the feed-to-egg ratio compared with that in the control group. Previous studies have reported that flavonoids and phenolic compounds in honeycomb contribute to maintaining gastrointestinal microbial balance, suppressing pathogenic bacteria and improving intestinal health and nutrient absorption, which could reasonably account for the reduced feed-to-egg ratio [20]. In addition to promoting laying performance, honeycomb supplementation also increased albumen weight and improved eggshell color, thickness, and weight, along with regulating egg pH. Flavonoid polyphenols have been shown to bind with ovalbumin, promote protein cross-linking, and form a denser gel structure [7]. Meanwhile, they may enhance albumen weight and stabilize pH by modulating pyruvate, methionine, and glutathione metabolism [8], and these effects are consistent with our findings. Furthermore, flavonoid polyphenols exhibit antioxidant capacity to reduce lipid peroxidation and oxidative stress. As a result, they can protect eggshell pigments such as protoporphyrin IX and support shell calcification during formation, ultimately improving eggshell quality [10].

The small intestine plays an essential role for nutrient absorption to support egg production in laying hens [21]. During the peak laying period, enhanced metabolism often elevates oxidative stress induced by excessive ROS generation [12]. Accumulating ROS then activate the TLR2/TLR4-MYD88 pathway, thereby amplifying the inflammatory response [13]. Notably, oxidative damage triggers intestinal inflammation and reduces nutrient absorption. In response to such oxidative stress, animals upregulate antioxidant enzyme activity and related genes to enhance the antioxidant capacity [15,22]. This study investigated the regulatory effect of dietary honeycomb supplementation on the intestinal antioxidant system in laying hens. The results showed that honeycomb upregulated the intestinal gene expression of *Nrf2*, *NQO1*, and *HO-1*, while downregulating *Keap1* expression. In line with the present findings, Keap1 promotes the ubiquitination of Nrf2 in the cytoplasm, resulting in the proteasomal degradation of Nrf2 [23]. Conversely, Nrf2, dissociating and subsequently translocating into the nuclear, binds to the antioxidant response element (NQO1, HO-1) and further promotes the expression of antioxidant enzyme genes [24,25], attenuating oxidative stress-induced intestinal mucosa damage and preserving the integrity of the intestinal epithelial barrier [26]. This finding suggests that honeycomb may modulate the Nrf2/Keap1 signaling pathway to strengthen cellular antioxidant capacity and maintain intestinal redox homeostasis. In addition, honeycomb increased the levels of antioxidant enzymes (GSH, SOD, and CAT) in the blood of laying hens and enhanced the expression of the heat shock protein *HSP60* in the intestine. Furthermore, serum antioxidant indicators were elevated, which indicated an improvement in antioxidant capacity [27]. Consequently, these findings suggest that honeycomb may alleviate oxidative stress and strengthen antioxidant defenses, which may be beneficial to intestinal function and nutrient utilization.

In addition, honeycomb supplementation significantly downregulated the intestinal gene expression of *TLR2*, *TLR4*, and *MYD88*. This indicates that honeycomb could attenuate the pro-inflammatory signaling cascade. Previous research has shown that Nrf2 activation suppresses oxidative stress-induced TLR4/MYD88/NF-κB signaling in intestinal epithelial cells and alleviates the intestinal inflammatory response [28,29]. Further analysis in the current study revealed that honeycomb significantly suppressed *TNF-α*, *IL-1β*, and *IL-2* expression, while elevating *IFN-γ* and *IL-10* expression. It is well known that the Nrf2/Keap1 pathway attenuates oxidative injury and inhibits pro-inflammatory cytokine production by negatively regulating the TLR4/MYD88 signaling cascade in laying poultry [30]. In addition, honeycomb downregulated the pro-apoptotic genes *BAX* and *P53*, while upregulating the anti-apoptotic gene *BCL-2*. This regulatory effect may be associated with enhancing antioxidant defense and suppressing TLR2/TLR4-MYD88 signaling. Collectively, these effects may improve nutrient utilization and further provide a material basis for yolk precursor synthesis in the liver.

Hepatic lipid metabolism is susceptible to dysregulation during nutrient imbalance or excessive energy intake, representing a key factor responsible for reduced egg production [31]. In laying hens, excessive hepatic TG synthesis causes massive lipid accumulation, damages normal liver function, impairs yolk precursor synthesis, and may trigger metabolic liver injury [32,33]. This injury is typically characterized by abnormal elevations in serum ALT and AST. In contrast, decreased serum ALT and AST levels reflect hepatocyte integrity and alleviated steatosis, thereby stabilizing hepatic function [34]. In the present study, honeycomb supplementation alleviated inflammatory infiltration of the liver and lipid droplet accumulation, while also markedly reducing serum AST, ALT and TG levels. Furthermore, the reduced serum TG levels were accompanied by enhanced expression of *VLDLR*, which mediates lipid uptake and delivery of yolk precursors to developing follicles [35]. These findings indicate that honeycomb may potentially alleviate hepatic injury by reducing the release of AST and ALT into the plasma, as well as regulating lipid supply for yolk formation. Although the hepatic histological observations were qualitative in nature, the serum biochemical parameters of hepatic yolk precursor synthesis further demonstrate that honeycomb exerts beneficial effects on liver function. Future quantitative histological analyses are required to further verify the hepatic morphological alterations observed herein.

To further investigate the effects of honeycomb on hepatic lipid synthesis, this study detected the expression of genes related to lipid metabolism. Acetyl-CoA carboxylase (*ACC*), sterol regulatory element-binding protein 1 (*SREBP1*), fatty acid synthase (*FAS*), and stearoyl-CoA desaturase 1 (*SCD1*) are involved in hepatic fatty acid synthesis and lipid deposition. Honeycomb supplementation significantly increased gene expression associated with fat synthesis (*ACC*, *SREBP1*, *FAS*, *SCD1*), as well as the expression of microsomal triglyceride transfer protein (*MTTP*) and peroxisome proliferator-activated receptor gamma (*PPAR-γ*). *FAS* and *SCD1* are involved in hepatic fatty acid synthesis in laying hens, and the fat content in poultry livers is positively correlated with the activity of *FAS* [36]. Interestingly, although lipid synthesis was enhanced, no obvious increase in hepatic lipid droplets was observed in the honeycomb-supplementation groups. This phenomenon may be attributed to the coordinated regulation of lipid synthesis, transport, and utilization. In laying hens, hepatic lipids are not only stored in the liver but also serve as essential precursors for yolk formation. Consistent with this physiological characteristic, honeycomb supplementation significantly increased *MTTP* and *PPAR-γ* expression. Specifically, both *MTTP* and *PPAR-γ* participate in fatty acid transport and degradation and effectively mitigate fat deposition in the liver [37,38], which suggests that honeycomb supplementation may promote hepatic lipid utilization and reduce excessive lipid accumulation.

Further analysis indicated that honeycomb supplementation significantly increased serum levels of FSH and E2. FSH regulates aromatase (*CYP19A1*) expression and enhances the biosynthesis of E2 in ovarian granulosa cells [39]. During egg yolk formation, E2 binds to estrogen receptors (ER). Subsequently, it regulates the activities of hepatic lipid metabolism-related enzymes (*FAS* and *SCD1*), promotes lipid metabolism and induces the synthesis of *ApoVLDL II* and *APOB* in the liver [40,41]. *APOB* further combines with triglycerides, cholesterol and phospholipids to promote the production of vitellogenin (*VTG*) and very low-density lipoprotein (*VLDL*) in the liver [9]. In the present study, honeycomb supplementation increased the serum levels of FSH and E2 and significantly upregulated the gene expression of *ApoVLDL II*, *APOB*, *VTG II* and *VLDLR* in the liver. Moreover, during oocyte formation, *VTG II*, the key yolk precursor subtype, is transported to the ovary along with hepatic *VLDL* via blood circulation [12], indicating that honeycomb could enhance hepatic lipid metabolism capacity, prevent abnormal hepatic lipid accumulation, and simultaneously promote the efficient transport of yolk precursors in laying hens. Consequently, improved hepatic lipid utilization and yolk precursor transport may provide sufficient substrates for follicular development and egg formation.

Follicular development and ovulation are regulated by the hypothalamus–pituitary–gonadal axis, depending on the secretion of GnRH, FSH, LH, and E2 [42]. Previous studies have demonstrated that elevated MLT concentration could promote the levels of FSH, LH, and E2, thus inducing ovulation [13,43]. FSH plays a key role in follicle growth and development and stimulates the secretion of P4 and E2. Meanwhile, increased circulating E2 levels can promote the synthesis of yolk precursors and their deposition in the ovaries [12,13]. In the current work, qualitative histological analysis of the ovaries suggested that honeycomb-supplemented hens exhibited a denser medullary structure and fewer atretic follicles. These changes were accompanied by markedly higher serum concentrations of FSH, E2, and MLT. In addition, the expression of *FSHR* and *LHR* were upregulated following honeycomb supplementation. Accordingly, the increased expression of these receptors may enhance the responsiveness of ovaries to FSH and LH, thereby amplifying the hormone mediated regulatory signals for follicular development. Accumulating evidence has shown that the increase in the *STAR* and *HSD3B1* genes promotes progesterone P4 synthesis [44]. *RUNX2* and *CYP17A1* are involved in mediating estrogen synthesis [45,46]. Consistent with the above results, honeycomb supplementation upregulated the expression of *CYP17A1*, *CYP19A1*, *HSD3B1*, and *HSD17B1*. Concurrently, genes associated with steroidogenesis (*FOXL2*, *STAR*, *SF1*, and *RUNX2*) were also significantly upregulated, thus further promoting steroid hormone synthesis. Collectively, these findings suggest that honeycomb supplementation could promote follicular development and accelerate yolk deposition through coordinated regulation of reproductive hormones, gonadotropin receptors and steroidogenic genes. Qualitative histological observations of the ovary were largely consistent with the above endocrine and molecular alterations. However, quantitative assessments of follicle counts and ovarian morphology are required to verify these histological outcomes in future studies.

From an applied perspective, honeycomb is a readily obtainable by-product of apiculture industry and costs considerably less than other bee-based products. The low dietary supplementation levels adopted in the present study (0.5–2.0 g/kg of diet) incur negligible extra feed expenditures. Moreover, the improved laying performance and feed conversion efficiency observed herein indicate promising economic advantages for commercial egg production. Nevertheless, comprehensive economic assessments under large-scale rearing systems remains necessary to verify its cost-effectiveness and practical applicability for commercial farms.

## 5. Conclusions

In this study, honeycomb was found to enhance intestinal antioxidant capacity and barrier function by the modulation of the Nrf2-Keap1 signaling pathway-related genes, improving nutrient absorption. Meanwhile, it synergistically regulates liver and ovarian functions, promotes yolk precursor synthesis and hormone secretion, facilitates follicular development, and ultimately improves the egg production performance of laying hens.

## Figures and Tables

**Table 1:** Composition and nutrient levels of the basal diet (air-dry basis) (%).

Ingredients	Content (%)	Nutritional Components	Content (%)
Corn	60.00	Total Calcium	3.76
Soybean Meal	25.00	Total Phosphorus	0.48
Premix	5.00	Crude Protein	15.68
Limestone Powder	8.00	Crude Fat	4.01
Phosphate Feed	1.00	Crude Fiber	3.52
Soybean Oil	1.00	Metabolic Energy (MJ/kg)	11.42
Total	100	Lysine	0.27
		Methionine	0.76
		Threonine	0.62

Description: Each kilogram of the premix contained vitamin A at 180–200 k IU/kg (source: retinyl acetate or retinyl palmitate), vitamin D_3_ at 60–100 k IU/kg (source: cholecalciferol; 1 IU = 0.025 μg cholecalciferol), vitamin E at ≥500 IU/kg (source: dl-α-tocopheryl acetate; 1 IU = 1 mg dl-α-tocopheryl acetate), vitamin K at 40–100 mg/kg, vitamin B_1_ at ≥40 mg/kg, vitamin B_2_ at ≥120 mg/kg, vitamin B_6_ at ≥36 mg/kg, vitamin B_12_ at ≥0.2 mg/kg, choline chloride at ≥7000 mg/kg, iron at 1400 mg/kg, copper at 200 mg/kg, zinc at 1200 mg/kg, manganese at 1200 mg/kg, iodine at 10 mg/kg, selenium at 4 mg/kg, calcium at 15%, total phosphorus at ≥2.4%, sodium chloride at 7%, and lysine at ≥3.1%; all values are measured values.

**Table 2:** Effects of honeycomb on the laying performance of hens.

Parameters	Groups				*p*-Value
	CON	H1	H2	H3	
AEW (g)	57.18 ± 0.26 ^c^	60.32 ± 0.16 ^a^	60.02 ± 0.3 ^ab^	59.46 ± 0.28 ^b^	<0.001
ADFI (g/d)	113.95 ± 2.15 ^b^	128.59 ± 2.71 ^a^	134.66 ± 3.23 ^a^	130.54 ± 3.22 ^a^	0.001
ALR (%)	88.39 ± 0.21 ^b^	91.16 ± 1.05 ^a^	93.39 ± 0.8 ^a^	91.22 ± 1.73 ^a^	<0.001
FCR	2.47 ± 0.03 ^b^	2.51 ± 0.04 ^b^	2.31 ± 0.03 ^a^	2.54 ± 0.04 ^b^	0.002

Description: CON, basal diet; H1, basal diet supplemented with 0.5 g/kg honeycomb; H2, basal diet supplemented with 1.0 g/kg honeycomb; H3, basal diet supplemented with 2.0 g/kg honeycomb. Values are presented as means ± SEM (*n* = 80). Values within different superscripts indicate significant differences (*p* < 0.05).

**Table 3:** Effects of honeycomb on egg quality in hens.

Parameters			Groups				*p*-Value
			CON	H1	H2	H3	
Egg Shape Index			1.3 ± 0.01	1.3 ± 0.01	1.29 ± 0.01	1.29 ± 0.01	0.849
Egg Shell Color L		L*	82.11 ± 0.64	80.75 ± 0.7	81.58 ± 0.63	80.26 ± 0.61	0.184
		A*	10.81 ± 0.36	11.7 ± 0.49	11.13 ± 0.44	11.97 ± 0.45	0.224
		B*	16.79 ± 0.7 ^b^	18.72 ± 0.77 ^ab^	18.06 ± 0.64 ^ab^	19.28 ± 0.62 ^a^	0.066
Egg Shell Strength (N)			38.01 ± 1.39	40.65 ± 1.44	37.4 ± 1.43	37.15 ± 1.39	0.280
Egg Shell Thickness (mm)			0.37 ± 0.004 ^b^	0.38 ± 0.004 ^a^	0.37 ± 0.004 ^b^	0.37 ± 0.004 ^ab^	0.018
Albumen Height (mm)			5.87 ± 0.2	6.17 ± 0.25	5.8 ± 0.23	6.1 ± 0.22	0.613
Yolk Weight (g)			18.31 ± 0.44	18.17 ± 0.34	18.46 ± 0.34	18.61 ± 0.37	0.855
Albumen Weight (g)			30.27 ± 0.64 ^b^	32.86 ± 0.54 ^a^	32.27 ± 0.49 ^a^	32.97 ± 0.72 ^a^	0.006
Egg Shell Weight (g)			7.8 ± 0.29 ^c^	8.17 ± 0.13 ^bc^	8.85 ± 0.13 ^a^	8.53 ± 0.14 ^ab^	0.001
Haugh Unit (HU)			76.44 ± 1.46	77.4 ± 1.64	74.56 ± 1.64	76.57 ± 1.82	0.661
PH	Albumen		8.7 ± 0.08 ^b^	8.9 ± 0.02 ^ab^	8.9 ± 0.01 ^a^	8.94 ± 0.02 ^a^	<0.001
	Yolk		6.43 ± 0.02 ^a^	6.39 ± 0.02 ^a^	6.35 ± 0.02 ^b^	6.41 ± 0.02 ^a^	0.022
	Whole Egg		6.86 ± 0.03	6.82 ± 0.03	6.82 ± 0.03	6.92 ± 0.03	0.062
Yolk Color			12.62 ± 0.09 ^a^	12.54 ± 0.11 ^a^	12.3 ± 0.15 ^a^	11.9 ± 0.15 ^b^	<0.001

Description: CON, basal diet; H1, basal diet supplemented with 0.5 g/kg honeycomb; H2, basal diet supplemented with 1.0 g/kg honeycomb; H3, basal diet supplemented with 2.0 g/kg honeycomb. L*, lightness value; A*, red-green chromatic value; B*, yellow-blue chromatic value (CIE Lab color space system). Values are presented as means ± SEM (*n* = 30). Within the same row, values with different superscript letters indicate significant differences (*p* < 0.05).

**Table 4:** Effects of honeycomb on serum antioxidant parameters and MDA levels in hens.

Parameters	Groups				*p*-Value
	CON	H1	H2	H3	
T-AOC (mM)	0.93 ± 0.12	0.92 ± 0.05	0.86 ± 0.12	0.81 ± 0.06	0.801
GSH (μmol/gprot)	6.12 ± 0.71 ^b^	6.35 ± 0.31 ^b^	5.57 ± 0.29 ^b^	9.3 ± 0.47 ^a^	0.002
SOD (U/ML)	16.38 ± 0.87 ^c^	19.63 ± 0.31 ^b^	21.54 ± 0.33 ^a^	20.01 ± 0.46 ^ab^	0.001
CAT (U/ML)	2.15 ± 0.29 ^c^	3.66 ± 0.32 ^b^	6.24 ± 0.41 ^a^	5.68 ± 0.25 ^a^	<0.001
MDA (nmol/ML)	1.14 ± 0.04	1.1 ± 0.27	0.76 ± 0.06	0.91 ± 0.06	0.275

Description: CON, basal diet; H1, basal diet supplemented with 0.5 g/kg honeycomb; H2, basal diet supplemented with 1.0 g/kg honeycomb; H3, basal diet supplemented with 2.0 g/kg honeycomb. Values are presented as means ± SEM (*n* = 3). Within the same row, values with different superscript letters indicate significant differences. T-AOC, total antioxidant capacity; GSH, glutathione; MDA, malondialdehyde; SOD, superoxide dismutase; CAT, catalase.

**Table 5:** Effects of honeycomb on serum biochemical parameters in hens.

Parameters	Groups				*p*-Value
	CON	H1	H2	H3	
ALT (U/L)	57.52 ± 5.75 ^a^	43.62 ± 1.31 ^b^	41.12 ± 1.56 ^b^	41.04 ± 2.73 ^b^	0.023
LPS (U/L)	5.01 ± 0.47	3.22 ± 0.31	4.11 ± 0.64	3.93 ± 1	0.353
AST (U/L)	50.22 ± 1.85 ^a^	53.16 ± 12.35 ^a^	38.74 ± 7.91 ^b^	31.26 ± 2.45 ^c^	<0.001
TG (mmol/L)	9.18 ± 0.61 ^a^	7.71 ± 1.04 ^ab^	6.04 ± 0.11 ^b^	6.57 ± 0.46 ^b^	0.037
T-CHO (mmol/L)	3.28 ± 0.74	3.9 ± 1.25	2.33 ± 0.19	3.11 ± 0.92	0.656
LDL-C (mmol/L)	2.05 ± 0.76	2.37 ± 0.78	2.83 ± 0.77	5.23 ± 1.37	0.158

Description: CON, basal diet; H1, basal diet supplemented with 0.5 g/kg honeycomb; H2, basal diet supplemented with 1.0 g/kg honeycomb; H3, basal diet supplemented with 2.0 g/kg honeycomb. Values are presented as means ± SEM (*n* = 3). Within the same row, values with different superscript letters indicate significant differences. ALT, alanine aminotransferase; AST, aspartate aminotransferase; LPS, lipopolysaccharide; TG, triglyceride; T-CHO, total cholesterol; LDL-C, low-density lipoprotein cholesterol.

## Data Availability

The original contributions presented in the study are included in the article; further inquiries can be directed to the corresponding authors.

## Supplementary Materials

The following supporting information can be downloaded at: https://www.mdpi.com/article/10.3390/ani16132016/s1. Table S1. Primers used for quantitative real-time PCR.

## Abbreviations

ROS	reactive oxygen species
ER-α	estrogen receptor alpha
ER	estrogen receptor
GnRH	gonadotropin-releasing hormone
AEW	average egg weight
ADFI	average daily feed intake
ALR	average laying rate
H1	0.5g/kg
H2	1.0g/kg
H3	2.0g/kg
P4	progesterone
FCR	feed conversion ratio
T-AOC	total antioxidant capacity
GSH	glutathione
MDA	malondialdehyde
SOD	superoxide dismutase
ALT	alanine aminotransferase
LPS	lipopolysaccharide
AST	aspartate aminotransferase
TG	triglyceride
T-CHO	total cholesterol
LDL-C	low-density lipoprotein cholesterol
FSH	follicle-stimulating hormone
PROG	progesterone
LH	luteinizing hormone
E2	estrogen
MLT	melatonin
*TLR2*	toll-like receptor 2
*TLR4*	toll-like receptor 4
*MYD88*	myeloid differentiation primary response 88
*Keap1*	Kelch-like ECH-associated protein 1
*Nrf2*	nuclear factor erythroid 2-related factor 2
*HO-1*	heme oxygenase-1
*NQO1*	NAD(P)H quinone dehydrogenase 1
*GPX4*	glutathione peroxidase 4
*SOD-1*	superoxide dismutase 1
*CAT*	catalase
*HSP60*	heat shock protein 60
*BAX*	Bcl-2-associated X protein
*P53*	tumor protein p53
*BCL-2*	B-cell lymphoma-2
*IFN-γ*	Interferon gamma
*TNF-α*	tumor necrosis factor-alpha
*IL-10*	interleukin-10
*IL-1β*	interleukin-1 beta
*IL-2*	interleukin-2
*COX-2*	cyclooxygenase-2
*PPAR-γ*	peroxisome proliferator-activated receptor alpha
*PPAR-α*	peroxisome proliferator-activated receptor gamma
*SREBP1*	sterol regulatory element-binding protein 1
*MTTP*	microsomal triglyceride transfer protein
*ACC*	acetyl-CoA carboxylase
*APOB*	apolipoprotein B
*FAS*	fatty acid synthase
*SCD1*	stearoyl-CoA desaturase 1
*ApoVLDLII*	apolipoprotein very low-density lipoprotein II
*VLDLR*	very low-density lipoprotein receptor
*VTG II*	vitellogenin II
*HSD17B1*	hydroxysteroid 17-beta dehydrogenase 1
*HSD3B1*	hydroxy-delta-5-steroid dehydrogenase, 3 beta- and steroid delta-isomerase 1
*CYP19A1*	cytochrome P450 family 19 subfamily A member 1
*CYP17A1*	cytochrome P450 family 17 subfamily A member 1
*LHR*	luteinizing hormone receptor
*FSHR*	follicle-stimulating hormone receptor
*ESR1*	estrogen receptor 1
*STAR*	steroidogenic acute regulatory protein
*SF1*	steroidogenic factor 1
*FOXL2*	forkhead box L2
*RUNX2*	runt-related transcription factor 2
