# Supplementary material for: Honeycomb Enhances the Egg-Laying Capacity of Laying Hens by Modulating Ovarian Function and Yolk Precursor Synthesis
Source: Animals (Basel). 2026 Jul 1;16(13):2016. doi: 10.3390/ani16132016 (PMC13360416; doi:10.3390/ani16132016)
Supplement: Supplementary file 1 [file animals-16-02016-s001.zip › animals-4381235-supplementary.pdf]

Table S1 Primers used for quantitative real-time PCR

Gene	Primer Sequence (5’ to 3’)	Accession No.	Product size/bp
GAPDH	F:ACTTTGGCATTGTGGAGGGT<br>R:GGACGCTGGGATGATGTTCT	NM_204305.2	121
IFN-γ	F:CTGACAAGTCAAAGCCGCAC<br>R:TCAAGTCGTTCATCGGGAGC	NM_205149.2	129
TNF-α	F:AAGCCCAGTGTGATCCTTCC<br>R:GCGAGCACTGCATTGTGTCT	XM_046900549.1	176
IL-10	F:ACAAAGCCATGGGGGAGTTC<br>R:TAGCGGACCGAACGTTAAGC	NM_001004414.4	190
IL-1β	F:GCCTGCAGAAGAAGCCTCG<br>R:GGAAGGTGACGGGCTCAAAA	NM_204524.2	210
IL-2	F:CACACCAACTGAGACCCAGG<br>R:CCGGTGTGATTTAGACCCGTA	NM_204153.2	174
COX-2	F:TTTTACGTACCTCGTGACTCC<br>R:CGTCCTCGTGCAGTCACATT	NM_001167718.2	265
Bcl-2	F:GATGACCGAGTACCTGAACC<br>R:CAGGAGAAATCGAACAAAGGC	NM_000633.3	114
BAX	F:TCCTCATCGCCATGCTCAT<br>R:CCTTGGTCTGGAAGCAGAAGA	XM_040676625.2	69
P53	F:CGGTCACCTGCACTTACTCC<br>R:TGGTCTCGTCGTCGTGGTAA	NM_205264.1	273
GPX4	F:CATCACCAACGTGGCGTCCAA<br>R:GCAGCCCCTTCTCAGCGTATC	NM_001346449.2	92
SOD-1	F:GGGAGGAGTGGCAGAAGTA<br>R:GCTAAACGAGGTCCAGCAT	NM_205064.2	162
CAT	F:AGCAGGTGCCTTTGGCTATT<br>R:TCCAGCAACAGTGGAGAACC	NM_001031215.2	121
HSP60	F:GTTTGACCGAGGCTACATC<br>R:CAAGACTAGAGTGCTGAGGG	NM_001012916.3	211
TLR2	F:CATTCACCATGAGGCAGGGATAG<br>R:GGTGCAGATCAAGGACACTAGGA	NM_001396826.1	157
TLR4	F:TGACCTACCCATCGGACACT<br>R:CTCAGGGCATCAAGGTCTCC	NM_001030693.2	171
MYD88	F:GATGATCCGTATGGGCATGGA<br>R:ATGGACCACACACACGTTCC	NM_001030962.5	170

Keap1	F:CATCAACTGGGTGCAGTACG<br>R:AGGGTGAGGTCCTGGAAGAT	MN416132.1	183
NQO1	F:AAGAAGAGAAGATTGAAGCGGCTGA<br>R:GCATGGCTTTCTTCTTCTGG	NM_001277620.2	171
Nrf2	F:CCACCCTAAAGCTCCATTCA<br>R:ATTCTTGCCTCTCCTGCGTA	NM_205117.1	217
HO-1	F:ATGCCTACACCCGCTATTTG<br>R:ATCTCAAGGGCATTCATTCG	NM_205344.1	178
PPAR-α	F:CAAACCAACCATCCTTGACGAT<br>R:GGAGGTCAGCCATTTTTTGGA	XM_046906371.1	65
PPAR-γ	F:GAATGCCACAAGCGGAGAAGGAG<br>R:TTTGGTCAGCGGGAAGGACTTTATG	NM_001001460.1	140
SREBP1F	F:GTCGGCGATCCTGAGGAA<br>R:CTCTTCTGCACGGCCATCTT	XM_046927254.1	105
MTTP	F:TTCAGGCATTCCGTGACCAAGTATG<br>R:TCCAACATTTCTGCTTTCCCTCTCC	NM_001109784.3	83
ACC	F:AGTGGATAACTGCTCAGATTGC<br>R:AGGGTTCATCTCCAGGGGTT	NM_205505.1	106
APOB	F:GCCGTTTGACTGGGAGTACA<br>R:TCTTCCCATTTCCTGGTGCC	NM_001044633.2	126
FAS	F:TGCTATGCTTGCCAACAGGA<br>R:ACTGTCCGTGACGAATTGCT	NM_205155.3	128
SCD1	F:CACCACCACTGTCACCTCAC<br>R:CCCAATAATGGCCCCTAGAT	NM_204890.2	214
ApoVLDL II	F:AGCAGGACAGCAGGTCTCTTGG<br>R:TCAGGGACAGTGGTGCTAAGGA	NM_205483.2	114
VLDLR	F:GTTCCTTCCTCATCCTCTTG<br>R:TCTCTTCACACCAGTTCCT	NM_205229.2	200
VTG II	F:TTGCAAGCTGATGAACACACAC<br>R:GATTGCTTCATCTGCCAGGTC	NM_001031276.2	192
HSD17B1	F:GCAGTGTTTGAGGTGAACGT<br>R:CATGTGGATGTTGAAGGGCC	NM_204837.1	216
HSD3B1	F:GGCTGCTGGACAAAGACTTC<br>R:GCCCAAGGTGTCAATGATGG	XM_025146591.3	173

CYP19A1	F:TGAGAGTTTGGATCAGCGGT<br>R:ACAAGACCAGGACCAGACAG	NM_001364699.2	220
CYP17A1	F:TGCTTCAACTCCTCCTACCG<br>R:CATCAGGTCCCTCACAGTGT	NM_001001901.3	243
LHR	F:GCAACGAATCGCTGACACTC<br>R:CTCTCAGGGCATCGTTGTGT	NM_204936.1	141
FSHR	F:TTAATTCCTGTGCTAACCCTTTCC<br>R:CCAAACTTGCTCAACAGAATGAAG	XM_040696841.2	85
ESR1	F:CTGGGCAAAGAGAGTTCCAG<br>R:GATTTCCACCATGCCCTCTA	NM_205183.2	196
STAR	F:TGCCTGAGCAGCAGGGATTTATCA<br>R:TGGTTGATGATGGTCTTTGGCAGC	NM_204686.3	148
SF1	F:GCGGGAGGAATAAGTT<br>R:ATGGCGTGGATGCTGT	NM_205077.2	169
FOXL2	F:CTGATCGCCATGGCCATACG<br>R:GGCGGATGCTGTTCTGCCA	NM_001012612.1	127
RUNX2	F:TAAAGGTGACGGTGGATGG<br>R:TGTGGATTAAAAGGACTTGGTG	XM_046912848.1	190
